# Dioscin pretreatment ameliorates ferroptosis in cardiomyocytes after myocardial infarction via inhibiting endoplasmic reticulum stress

**DOI:** 10.1186/s10020-025-01102-y

**Published:** 2025-01-29

**Authors:** Chang Wu, Xueping Shen, Pan Lou, Dongyan Song

**Affiliations:** 1 The First People’s Hospital of Lin’an District, No. 360, Yikang Street, Jinnan Subdistrict, Lin’an District, Hangzhou, Zhejiang 311300 China; 2 Wenzhou Medical University, Wenzhou, Zhejiang 325000 China; 3 Center of Prenatal Diagnosis Huzhou Maternity & Child Health Care Hospital, Huzhou, Zhejiang 313000 China; 4 The Second People’s Hospital of Anji, Huzhou, Zhejiang 313307 China

**Keywords:** Dioscin, Ferroptosis, Endoplasmic reticulum stress, Myocardial infarction

## Abstract

**Background:**

Myocardial infarction (MI) remains a leading cause of mortality globally, often resulting in irreversible damage to cardiomyocytes. Ferroptosis, a recently identified form of regulated cell death driven by iron-dependent lipid peroxidation, has emerged as a significant contributor to post-MI cardiac injury. The endoplasmic reticulum (ER) stress response has been implicated in exacerbating ferroptosis.

**Methods:**

Here, we investigated the potential of Dioscin, a natural compound known for its diverse pharmacological properties, in mitigating ferroptosis in cardiomyocytes following MI by targeting ER stress.

**Results:**

In animal models subjected to MI, administration of Dioscin notably improved cardiac function, reduced infarct size by approximately 24%, and prevented adverse remodeling, highlighting its therapeutic potential. Through in vitro and in vivo models of MI, we demonstrated that Dioscin treatment significantly attenuates ferroptosis in cardiomyocytes, as evidenced by a decrease in lipid peroxidation by about 19% and preserved mitochondrial integrity. Moreover, Dioscin exerted its protective effects by inhibiting ER stress markers, such as the phosphorylation levels of PERK and eIF2α proteins, and the expression levels of BIP and ATF4 proteins, thus disrupting the ER stress-mediated signaling cascade associated with ferroptosis.

**Conclusion:**

Overall, our findings suggested that Dioscin holds promise as a therapeutic agent against post-MI cardiac injury by mitigating ferroptosis via the suppression of ER stress. Further investigations into the precise molecular mechanisms and clinical translation of Dioscin’s cardioprotective effects are warranted, offering a potential avenue for novel therapeutic interventions in MI-related cardiac complications.

**Graphical Abstract:**

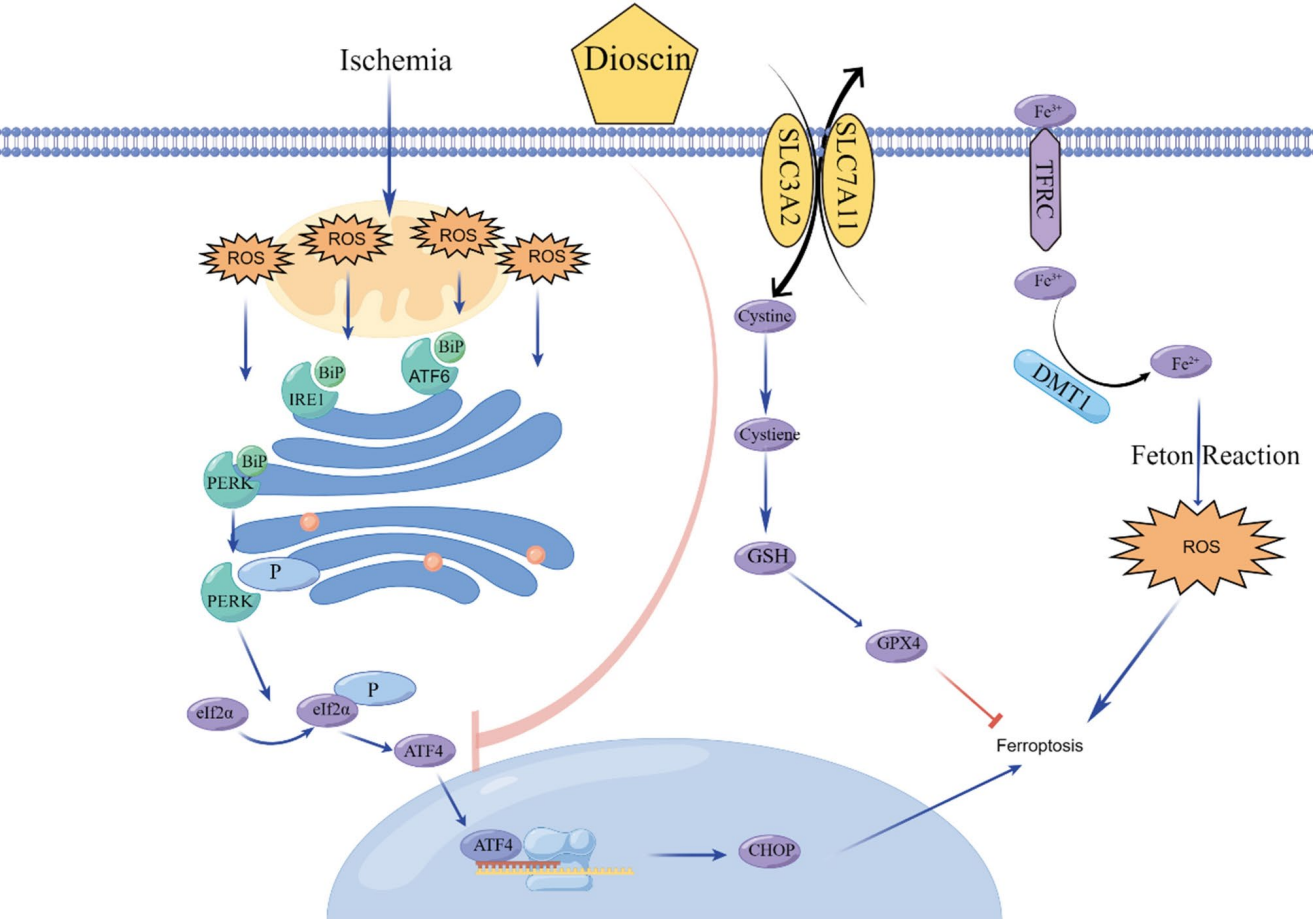

## Introduction

A growing number of cardiovascular disease risk factors are becoming more prevalent due to population aging and unhealthy lifestyles (Kotseva et al. 2016; Pasha et al. 2020). In 2022, the age-standardized prevalence of cardiovascular diseases across different regions ranged from 5,881.0 per 100,000 in South Asia to 11,342.6 per 100,000 in Central Asia (Mensah et al. 2023). The age-standardized mortality rate ranged from 73.6 per 100,000 in the high-income Asia-Pacific region to 432.3 per 100,000 in Eastern Europe. Globally, ischemic heart disease had the highest age-standardized disability-adjusted life years (DALYs) among all diseases, reaching 2,275.9 per 100,000. Myocardial infarction (MI) stands as the leading cause of cardiovascular disease-related deaths (Reed et al. 2017). While reperfusion therapy has reduced mortality rates associated with MI, the risk of chronic heart failure and/or death in MI patients after discharge continues to rise (Benjamin et al. 2017; Martinet et al. 2019; Raziyeva et al. 2022; Schafer et al. 2022). Further reducing myocardial cell death caused by MI and preserving more functioning myocardium is crucial for improving the prognosis of MI patients. Therefore, effective therapies must be developed as soon as possible to prevent cardiomyocyte death in MI and maintain normal cardiac function (Makover et al. 2022).

The process of ferroptosis is a type of regulated cell death caused by the oxidation of lipids and the accumulation of ferrous iron that is tightly correlated with numerous biological processes in cells (Li et al. 2022; Miyamoto et al. 2022). Polyunsaturated fatty acids are involved in creating this ancient vulnerability, in different environments, cells have evolved complex systems to defend against this vulnerability. Glutathione peroxidase 4 (GPX4) is an antioxidant enzyme that often exhibits dysfunction, and its active state is a critical mechanism regulating ferroptosis (Ursini and Maiorino 2020). Recent studies have found that the expression of GPX4 is significantly decreased in the early and middle stages of MI, suggesting that MI may lead to ferroptosis in myocardial cells (Pabisz et al. 2022). Additionally, ferroptosis often triggers inflammation, exacerbating post-MI heart failure and adverse myocardial remodeling (Komai et al. 2022). Both *in vivo and in vitro* experiments have shown that antioxidants or iron chelators can inhibit ferroptosis and improve cardiac function after MI in animals (Petrovic et al. 2018; Liu et al. 2022a; Shen et al. 2022b). Therefore, inhibiting ferroptosis in myocardial cells may represent a novel approach for treating MI and improving cardiac function (Liu et al. 2021; Komai et al. 2022).

The endoplasmic reticulum (ER) is the most critical site of lipid peroxidation during ferroptosis (Stockwell 2022). Under normal circumstances, nascent proteins are folded and processed by ER (de la Calle et al. 2022; Liu et al. 2022b). When ER function is disturbed, misfolded proteins accumulate in the ER, leading to ER stress. To restore ER homeostasis, the ER activates a defense mechanism called the unfolded protein response (UPR) (Daniels Gatward et al. 2022). However, if the cell fails to regain homeostasis, cell death is triggered (Sano and Reed 2013). Studies have shown that the ferroptosis inducer Erastin can induce significant ER stress by activating the eukaryotic translation initiation factor 2 A (EIF2A)/activating transcription factor 4 (ATF4) pathway (Dixon et al. 2014). Furthermore, ATF4-mediated upregulation of HSPA5 expression prevents the degradation of GPX4, thereby increasing resistance to cellular ferroptosis (Zhu et al. 2017). The upregulation of ATF4-mediated membrane transporter protein SLC7A11 is also associated with resistance to ferroptosis (Chen et al. 2017). In summary, ER stress may play a role in myocardial cell ferroptosis, and further exploration of the mechanisms involved is warranted.

Dioscin, a natural product found in several medicinal plants including Dioscorea and Liliaceae, is widely used as an antimicrobial agent (Wang and Wang 2022). It has been shown in numerous studies that Dioscin is beneficial to the cardiovascular system (Bao et al. 2022), immune system (Jin et al. 2022; Mao et al. 2022), and cancer, as well as having antioxidant or anti-oxidant properties (Shang et al. 2022; Shen et al. 2022a; Zhong et al. 2022). Our purpose of the study was to determine whether Dioscin affects cardiac injury and dysfunction induced by MI, as well as investigate whether ferroptosis and ER stress play a role in Dioscin’s effects.

## Materials and methods

### Animal procedures

Forty male C57BL/6 mice (aged 6–8 weeks, weighing 18–22 g) were purchased through the Experimental Animal Center of Wenzhou Medical University from the Zhejiang Vital River Laboratory Animal Technology Co. A 12-hour light/dark cycle was applied to mice from different experimental groups housed in cages. Mice were housed in a standard facility under specified pathogen-free conditions with access to ad libitum water and food. Mice were monitored daily for health and activity. All experimental procedures were carried out according to the instructions of the United States National Institutes of Health Guide for the Care and Use of Laboratory Animals and approved by the Laboratory Animal Ethics Committee of Wenzhou Medical University (Ethics Approval Number: wydw2022-0079).

### Animal grouping and pretreatment

All mice were divided into three groups randomly: (a) Mice in the Sham group underwent sham operation; (b) An MI intervention was administered to the MI group of mice; (c) Mice in the Dioscin group were pretreated with Dioscin (40 mg/kg/day) for 2 weeks before they were treated with MI. The Dioscin was dissolved in 0.5% CMC-Na solution before use and injected intraperitoneally.

### MI model

As previously described (Wu et al. 2023), mice were anesthetized with isoflurane and then intubated. Afterward, the third and fourth intercostal spaces were opened to allow access to the heart. The left anterior descending coronary artery (LAD) was localized using the left atrial appendage. The LAD is closed with a 7 − 0 silk suture. The heart was returned to its original position, then the thoracic cavity was drained of air. The skin incision is closed with a 4 − 0 silk suture. As part of the recovery process, the mice were placed on an electric blanket and closely observed. The sham-operated group underwent the same procedure but without ligation.

Seven days after MI, echocardiography (ECHO) was performed to evaluate cardiac function in mice. On the following day after the ECHO, euthanasia was conducted via intraperitoneal injection of sodium pentobarbital (100 mg·kg^− 1^). After confirming death, the chest cavity was opened to access the heart. Blood samples were collected through cardiac puncture and centrifuged at 3,000 rpm for 15 min to separate the serum. The heart was excised and rinsed with cold phosphate-buffered saline (PBS) for subsequent analysis.

### Cardiac functional assessment

Seven days post-MI modeling, cardiac function in mice (*n* = 6) was assessed using the Vevo2100 ultra-high-resolution small animal ultrasound imaging system (including software) (VisualSonics, Canada). A panoramic view of the heart was obtained through B-mode ultrasound, followed by M-mode ultrasound to capture real-time images of cardiac structures along the sampling line. The following parameters were measured: left ventricular ejection fraction (LVEF), left ventricular fractional shortening (LVFS), left ventricular internal diameter in diastole (LVIDd), and left ventricular internal dimension in systole (LVIDs).

Serum was separated by centrifugation at 3,000 rpm for 15 min. Creatine kinase MB (CK-MB) isoenzyme assay kit (Nanjing Jiancheng, China) was used to measure serum CK-MB, enzyme-linked immunosorbent assay (ELISA; E-EL-M1801c, Elabscience, Wuhan, China) was used to measure serum cTnT concentration, and serum LDH activity was detected using lactate dehydrogenase (LDH) assay kit (Nanjing Jiancheng, China).

### MI size detection

MI was visualized by Triphenyltetrazolium chloride (TTC, Sigma, USA) staining. The hearts were harvested, briefly cleaned and embedded in optimal cutting temperature compound (OCT), frozen at -80 °C, and sectioned. A 1% TTC staining solution was applied to the heart slices for 15 min and then fixed in 4% paraformaldehyde for 24 h. Staining showed a red color in the normal tissue areas and a pale white color in the infarcted areas.

### Histological analysis

Sections were prepared by fixing heart tissue with 4% paraformaldehyde solution, followed by paraffin embedding. The sections were stained with hematoxylin-eosin (H&E) and Masson’s staining. Staining was conducted using H&E staining kit (Solarbio, China) and trichrome stain kit (Solarbio, China) according to the instructions provided by the manufacturer. Microscope images were taken by Olympus. Masson staining showed blue collagen fibers, mucus, and cartilage, red cytoplasm, muscle, fibrin, and neuroglia, and black-blue nuclei.

### Prussian blue staining

Frozen tissue was cut into thin slices, dropped into Perls Stain (G1426, Solarbio, China) and, completely covered with tissue. The tissue was incubated at 37 °C for 30 min and rinsed twice with tap water for 2 min each time. It was counterstained with Perls Counterstain Solution for 30 ~ 60 s, washed, dried, and observed under the microscope. The results showed a blue color for the iron ions and a red color for the cell nuclei.

### Myocardial tissue and Cellular immunofluorescence

Frozen tissue or cell sections that had been fixed in paraformaldehyde were immersed in 0.3% Triton solution for 10 min. They are then blocked with 5% BSA for 1 h at room temperature and incubated overnight with the corresponding primary antibody. The following day, the secondary antibody was incubated for 1 h in the dark and the nuclei were stained with DAPI. As soon as the solution was quenched with anti-fluorescence quenching solution, pictures were taken under a microscope (Leica, Germany).

### Oxygen and glucose deprivation (OGD) model

H9C2 cells were used to simulate the OGD model. The cells were cultured hypoxia (5% CO_2_, 94% N_2_, and 1% O_2_) in serum-free medium for 24 h. Dioscin (5 µM) was applied to the cells for 24 h before placing them in the hypoxic incubator. Dioscin was dissolved with 0.1% dimethyl sulfoxide (DMSO) before use for all in vitro experiments.

### Cell viability assay

The number of viable cells of treated H9C2 cells in 96-well plates was measured using the Cell Counting Kit-8 (CK04, Dojindo, Japan) according to the instructions.

### Hoechst 33,342/PI staining assay

H9C2 cells were cultured in 6 well plates and rinsed them with phosphate buffered saline (PBS). Afterwards, Hoechst 33,342 and PI were added as directed by the manufacturer (Beyotime, China). Finally, mounting medium were added to prevent fading.

### Western blot analysis

Proteins of myocardial tissue/cell samples were extracted with phosphatase and protease inhibitors in RIPA buffer. Protein preparation and concentration determination kits were purchased from Beyotime (Shanghai, China). Proteins of different molecular weights were separated using SDS-PAGE gels, transferred to 0.22 μm PVDF membranes, then blocked with skimmed milk powder and incubated with primary antibodies of TFRC (A5865, ABclonal), SLC7A11 (A2413, ABclonal), GPX4 (A1933, ABclonal), p-PERK (AP1420, ABclonal), PERK (A18196, ABclonal), BIP/GRP78 (A0241, ABclonal), ATF4 (A0201, ABclonal), p-eIf2α (AP0692, ABclonal), eIF2α (A0764, ABclonal) at 4℃ overnight. The next day, protein bands after incubation with the corresponding secondary antibodies were scanned using a ChemiDoc MP device (Bio-Rad, USA).

### RNA extraction and qPCR

Total RNA was extracted using TRIzol reagent (Takara, China) according to the instructions. RNA was reversed transcribed using a HiScript II Q RT SuperMix (R233-01, Vazyme, Nanjing China). qPCR was performed with Taq Pro Universal SYBR qPCR Master Mix (Vazyme, China). β-actin was used as a housekeeping gene. The primers (5′ to 3′) utilized in this study included mouse TFRC (forward, GTTTCTGCCAGCCCCTTATTAT; reverse, GCAAGGAAAGGATATGCAGCA), SLC7A11 (forward, CAGTTGTGGCCACCATCTCC; reverse, CGCTGCCCGTGTTCTGGAGT), GPX4 (forward, TGTGCATCCCGCGATGATT; reverse, CCCTGTACTTATCCAGGCAGA), and β-actin (forward, GTGACGTTGACATCCGTAAAGA; reverse, GCCGGACTCATCGTACTCC). The 2^−ΔΔCT^ method was used to calculate the relative gene expression levels of each target gene.

### ROS detection

ROS fluorescence was measured by staining DHE/DCFH-DA (Beyontime, China). In accordance with the instructions provided by the manufacturer, DHE/DCFH-DA working solution was added after rinsing the cells with sterile PBS. Afterwards, the cells were incubated at 37 °C under 5% CO_2_ for 30 min. After washing the cells with PBS three times, images were taken with an Olympus microscope.

### Monitoring oxidative stress levels

According to the manufacturer’s protocol, malondialdehyde (MDA), glutathione (GSH), superoxide dismutase (SOD) content in the heart and H9C2 cells were measured using commercial kits (Nanjing Jiancheng Bioengineering Institute, Nanjing, China).

### The determination of Ferrous Iron

Cell Ferrous Iron Colorimetric Assay Kit (E-BC-K881-M, elabscience, Wuhan, China) was used to measure cellular Fe^2+^ contents. Briefly, H9C2 cells were seeded into plates and cultured overnight. Adherent cells were washed away with trypsin containing 0.25% EDTA. The cells are counted at 10^6^ cells per well using Countstar and lysed on ice. The cells were centrifuged at 15,000 rpm for 10 min at 4 °C. The supernatant was collected and working reagents were added as directed. Absorbance was measured at 593 nm using a microplate reader.

### Analysis of statistical

Data were presented as mean ± standard deviation, and were analyzed using Graph-Pad Prism 8. Normally distributed data were used in all analyses. In order to compare two experimental groups, a two-tailed unpaired Student’s t-test or an analysis of variance, followed by a Duncan’s T3 multiple-range test, was conducted. *P* < 0.05 were considered as statistically significant.

## Results

### Dioscin pretreatment ameliorated deterioration of cardiac function in MI mice model

**Fig. 1:**
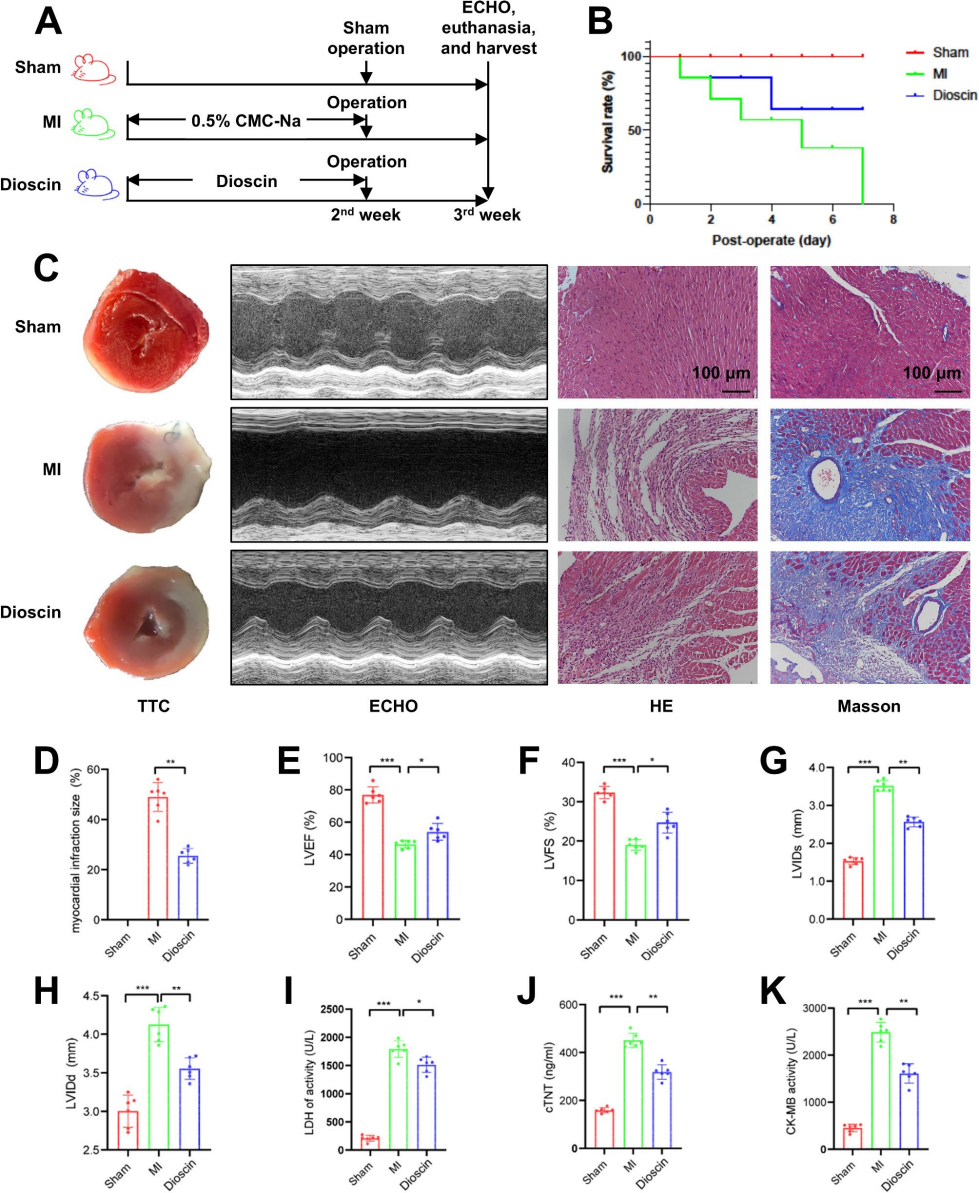
The effects of Dioscin pretreatment on the survival rate and heart function in a mouse MI model. (**A**) Flowchart of Dioscin administration (*n* = 10). (**B**) The survival rate of mice (*n* = 10). (**C**) Representative TTC, ECHO, **H**&**E**, and Masson staining images. (**D**) Quantification of the MI area (*n* = 6). (**E**-**H**) Echocardiographic parameters, including LVEF (**E**), LVFS (**F**), LVIDs (**G**), and LVIDd (**H**) (*n* = 6). (**I**-**K**) Cardiac injury markers, including LDH (**I**), cTNT (**J**), and CK-MB (**K**) levels (*n* = 6). ^*^P < 0.05, ^**^P < 0.01, ^***^P < 0.001. MFI, mean fluorescence intensity

The whole process of MI mice model establishment and intervention is shown in Fig. 1A. The survival rate of mice pretreated with Dioscin after MI surgery was significantly higher than that of the MI group (Fig. 1B). We next assessed myocardial infarct size as a measure of the effect of Dioscin pretreatment on MI. TTC staining showed that Dioscin pretreatment inhibited the expansion of the MI area in MI mice (Fig. 1C-D). To confirm the effect of Dioscin pretreatment on structural changes in MI hearts, cardiac pathologic changes were assessed by H&E staining and Masson staining. Histological assessments of the experimental animals revealed regular patterns of myocardial fiber arrangement and clear structures in the Sham group. Increased leukocyte infiltration, irregular myocardial fibers, and collagen deposition were noted in the MI group. Dioscin pretreatment reduced these pathologic alterations noted in the MI group. To investigate the effect of Dioscin pretreatment on the cardiac function of mice, using M-mode ECHO to measure cardiac parameters. Mice with MI showed impaired cardiac function, which was demonstrated by significant decreases in LVEF and LVFS, as well as increases in LVIDd and LVIDs. The application of Dioscin pretreatment significantly improved the cardiac contractile function (Fig. 1E-H). In addition, the levels of cTnT, CK-MB, and LDH were reduced by Dioscin pretreatment significantly after MI (Fig. 1I-K). Taken together, our results suggested that Dioscin pretreatment ameliorates the structural and functional damage of the heart caused by MI, thus exerting a protective effect on the MI model.

### Dioscin pretreatment reduced ferroptosis in cardiomyocytes while MI

**Fig. 2:**
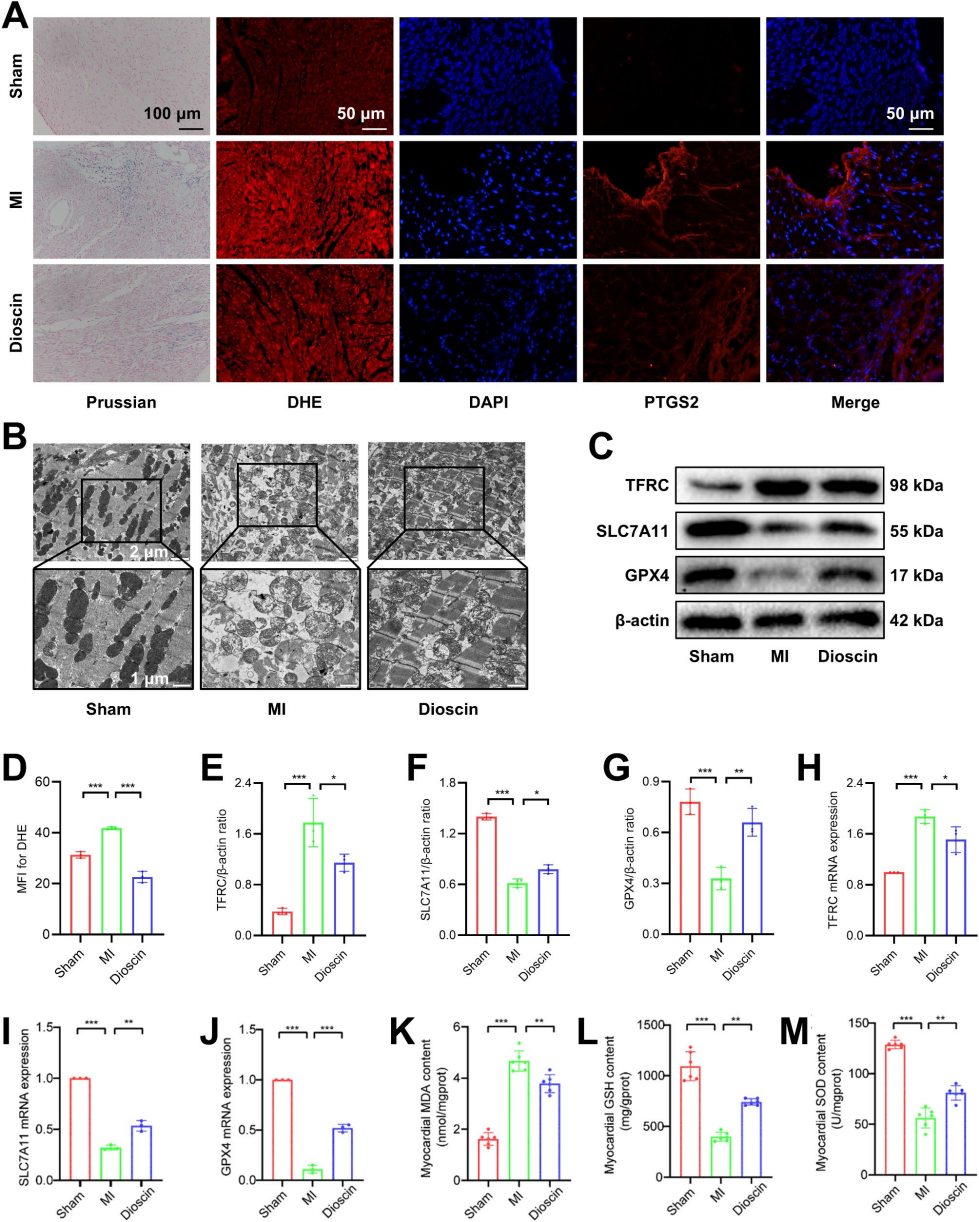
Dioscin pretreatment reduced ferroptosis in cardiomyocytes during MI. (**A**) Representative Prussian blue, DHE, and PTGS2 immunofluorescence staining images. (**B**) Mitochondrial morphology. (**C**) Representative Western blot images of ferroptosis markers. (**D**) Quantification of DHE fluorescence intensity (*n* = 3). (**E**-**G**) Quantification of TFRC (**E**), SLC7A11 (**F**), and GPX4 (**G**) proteins expression (*n* = 3). (**H**-**J**) mRNA levels of TFRC (**H**), SLC7A11 (**I**), and GPX4 (**J**) were detected by qPCR (*n* = 3). (**K**-**M**) MDA (**K**), GSH (**L**), and SOD (**M**) levels (*n* = 6). ^*^P < 0.05, ^**^P < 0.01, ^***^P < 0.001

Next, we investigated the anti-ferroptosis effects of Dioscin pretreatment in the MI mice model. Using Prussian blue staining, we found that the hearts of mice in the MI group had a large amount of iron deposition compared with those in the Sham group, whereas iron deposition was significantly reduced in mice pretreated with Dioscin (Fig. 2A). Mitochondrial ROS production was assessed by dihydroethidium (DHE) staining. Mice in the MI group showed a significant increase in DHE staining (red fluorescence), and Dioscin pretreatment partially quenched the MI-induced increase in DHE staining (Fig. 2D). Immunofluorescence results showed that PTGS2, a gene encoding prostaglandin endoperoxide synthase 2, which is a biomarker of iron death, was elevated during MI, whereas Dioscin-pretreated mice showed reduced levels of expression compared with MI mice. In addition, changes in cellular mitochondria were assessed by transmission electron microscopy (TEM), which showed smaller mitochondria, increased membrane density, and reduced cristae in the MI group of mice, and restoration of the altered mitochondrial ultrastructure in Dioscin-pretreated MI mice (Fig. 2B). We investigated the expression levels of ferroptosis proteins in the heart tissues and found that TFRC was upregulated and SLC7A11 and GPX4 were downregulated during MI, whereas in Dioscin pretreated mice the expression levels of TFRC were reduced and SLC7A11 and GPX4 were increased when compared with MI mice (Fig. 2C and E-G). At the same time, we also detected the mRNA transcription level of TFRC, SLC7A11, and GPX4, and the variety was consistent with the protein expression levels (Fig. 2H-J). Pretreatment of Dioscin further reduced MDA levels, and increased GSH and SOD levels in myocardial cells (Fig. 2K-M). The above results suggested that Dioscin pretreatment promotes resistance to ferroptosis in MI mice.

### Dioscin pretreatment inhibited cardiomyocyte ER stress in the MI model

**Fig. 3:**
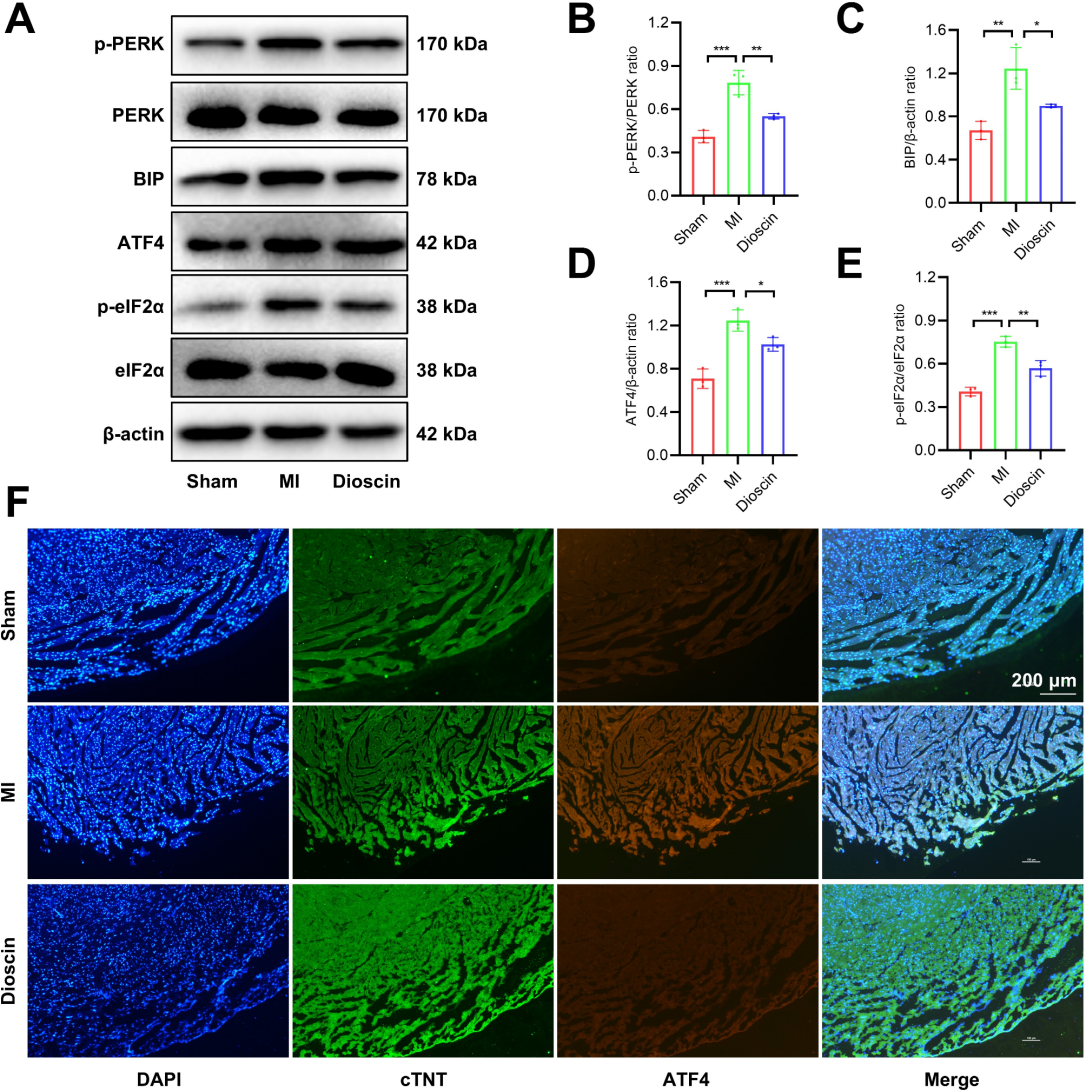
Dioscin pretreatment inhibited cardiomyocyte ER stress in the MI model. (**A**) Representative Western blot images of ER stress markers. (B-E) Quantification of PERK phosphorylation (**B**), GRP78/BIP (**C**), ATF4 (**D**), and eIF2α phosphorylation (**E**) expression (*n* = 3). (**F**) Representative double immunofluorescent staining images of ATF4 and cTNT. ^*^P < 0.05, ^**^P < 0.01, ^***^P < 0.001

Recent studies have shown that ER stress can regulate the ferroptosis process (Zhao et al. 2021; Xu et al. 2023). To further elucidate the potential mechanism of ferroptosis inhibition by Dioscin pretreatment, protein levels of ER stress-related markers were examined by western blot analysis. We found that the phosphorylation levels of PERK and eIF2α proteins as well as ATF4 and GRP78/BIP protein expression were up-regulated during MI, and their expression levels were partially restored in Dioscin pretreated mice (Fig. 3A-E). Fluorescence staining showed an increase in ATF4 fluorescence in MI tissue and a decrease in Dioscin-pretreated mice MI tissue (Fig. 3F).

### Dioscin pretreatment reduced ferroptosis in H9C2 cells in the OGD model

**Fig. 4:**
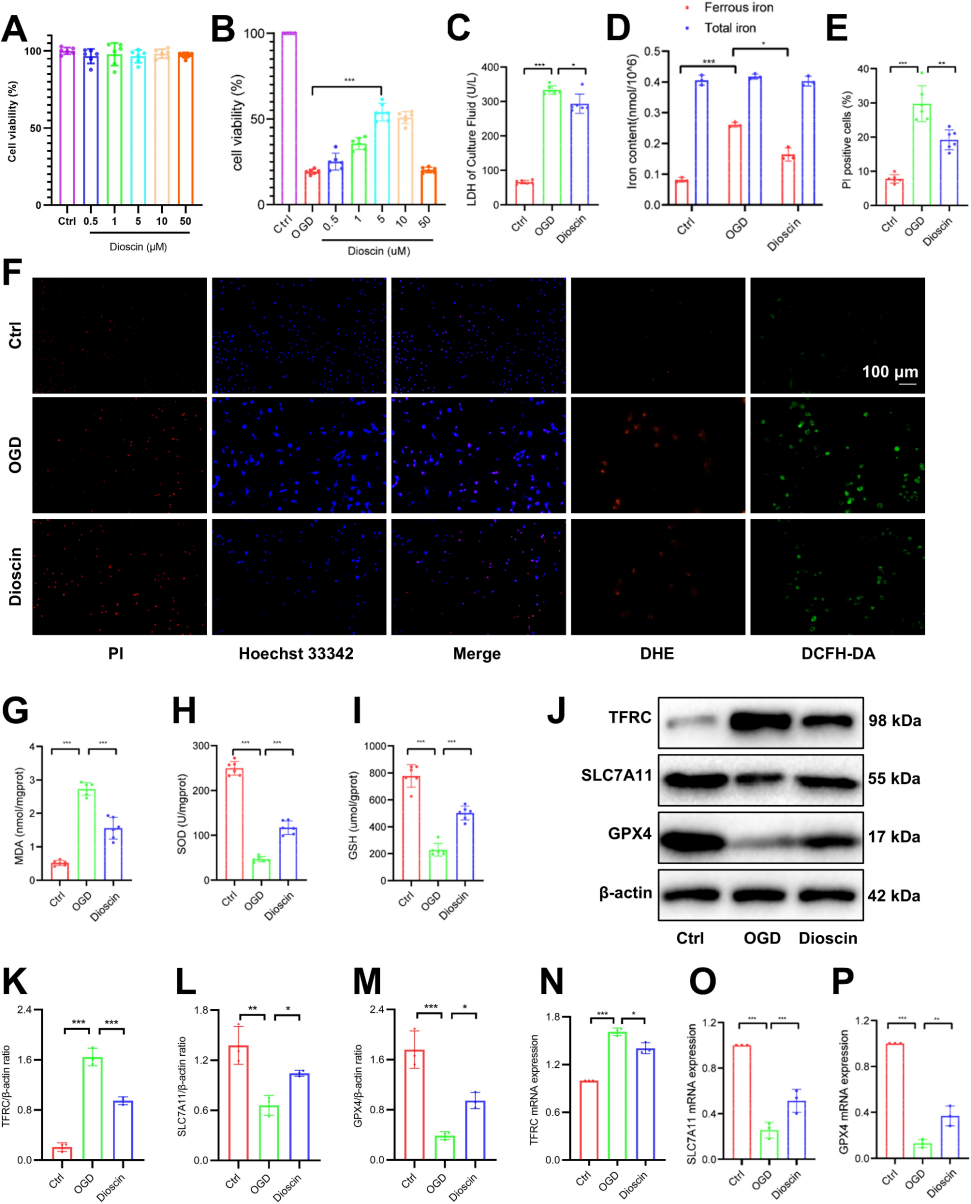
Dioscin pretreatment reduced ferroptosis in H9C2 cells under OGD. (**A**) Toxicity assay of different concentrations of Dioscin in H9C2 cells (*n* = 6). (**B**) Survival rate of H9C2 cells pretreated with different concentrations of Dioscin under OGD conditions (*n* = 6). (**C**) LDH levels (*n* = 6). (**D**) Fe^2+^ and total iron content (*n* = 6). (**E**) PI staining positive ratio (*n* = 6). (**F**) Representative PI staining, DHE, and DCFH-DA staining images. (**G**-**I**) MDA (**G**), SOD (**H**), and GSH (**I**) levels (*n* = 6). (**J**) Representative Western blot images of ferroptosis markers. (K-M) Quantification of TFRC (**K**), SLC7A11 (**L**), and GPX4 (**M**) proteins (*n* = 3). (**N**-**P**) mRNA levels of TFRC (**N**), SLC7A11 (**O**), and GPX4 (**P**) were detected by qPCR (*n* = 3). ^*^P < 0.05, ^**^P < 0.01, ^***^P < 0.001

Based on previous studies (Yao et al. 2017; Liu et al. 2020; Xu et al. 2021; Zhang et al. 2022c), we established a series of concentration gradients to determine the optimal concentration of Dioscin for pre-treating H9C2 cells. As shown in Fig. 4A, Dioscin exhibited no significant cytotoxicity toward H9C2 cells within 24 h at concentrations of 0.5, 1, 5, 10, and 50 µM. According to Fig. 4B, the activity of H9C2 cells first increased and then decreased with the increase of pretreated Dioscin concentration. Therefore, we selected a concentration of 5 μm for the in vitro test. We assessed the extent of cell damage by measuring LDH in the supernatant of the cell culture medium, and we found that Dioscin pretreatment also improved OGD injury in vitro (Fig. 4C). We measured ferrous iron concentrations and total iron concentrations at the cellular level, and Dioscin pretreatment inhibited the increase in ferrous iron in the OGD model, whereas total iron remained relatively unchanged (Fig. 4D). Based on PI staining, higher survival rates were observed in OGD cells pretreated with Dioscin (Fig. 4E-F). Dioscin pretreatment rescued the elevated MDA levels and decreased GSH and SOD levels in the OGD model (Fig. 4G-I), reflecting its effective antioxidant activity (Zhang et al. 2022c). We investigated the mRNA and protein expression levels of ferroptosis-related genes in the OGD model and found that TFRC was up-regulated and SLC7A11 and GPX4 were down-regulated in the OGD model, whereas in the Dioscin pretreated mice, the expression level of TFRC was reduced and SLC7A11 and GPX4 were increased relative to the OGD model (Fig. 4J-P).

### Dioscin pretreatment inhibited ER stress in H9C2 cells in the OGD model

#### Coi

**Fig. 5:**
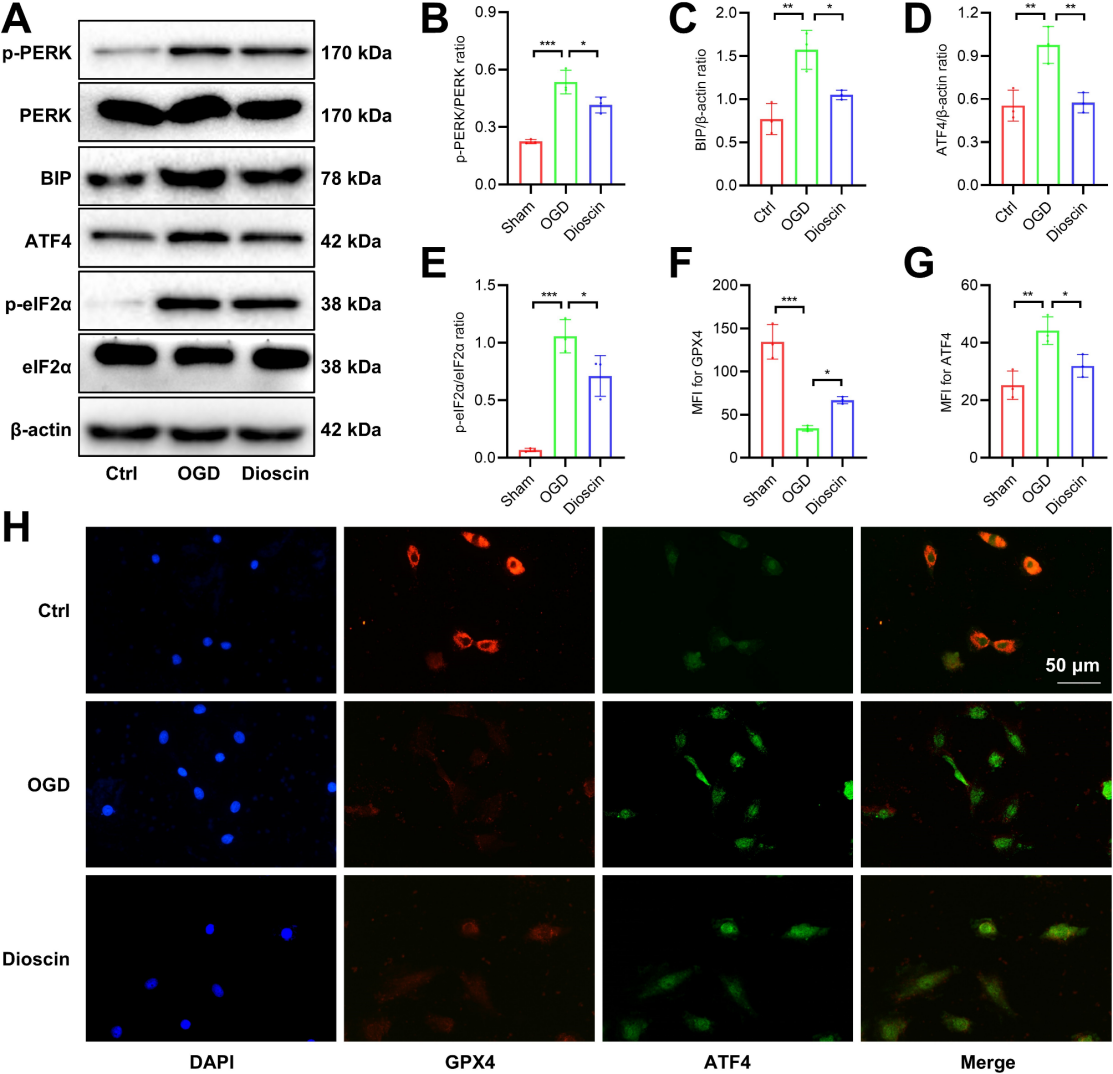
Dioscin pretreatment inhibited ER stress in H9C2 cells under OGD. (**A**) Representative Western blot images of ER stress markers. (**B**-**E**) Quantification of PERK phosphorylation (**B**), GRP78/BIP (**C**), ATF4 (**D**), and eIF2α phosphorylation (**E**) expression (*n* = 3). (**F**) Quantification of GPX4 fluorescence intensity (*n* = 3). (**G**) Quantification of ATF4 fluorescence intensity (*n* = 3). (**H**) Representative immunofluorescent staining images of GPX4 and ATF4. ^*^P < 0.05, ^**^P < 0.01, ^***^P < 0.001. MFI, mean fluorescence intensity

ncidentally, Dioscin pretreatment also improved the ER stress of the OGD model in vitro. Due to sugar and oxygen deprivation, the phosphorylation levels of PERK and eIF2 proteins as well as ATF4 and GRP78/BIP protein expression obviously increased, However, the expression of these proteins in the group pretreated with Dioscin is not so high as that in the OGD group (Fig. 5A-E), and the fluorescence staining showed an increase in ATF4 fluorescence in OGD and a decrease in Dioscin-pretreated group compared with the OGD group, as in the results of tissue fluorescence (Fig. 5F-H).

### Dioscin pretreatment inhibited ferroptosis in OGD via suppressing ER stress in vitro

**Fig. 6:**
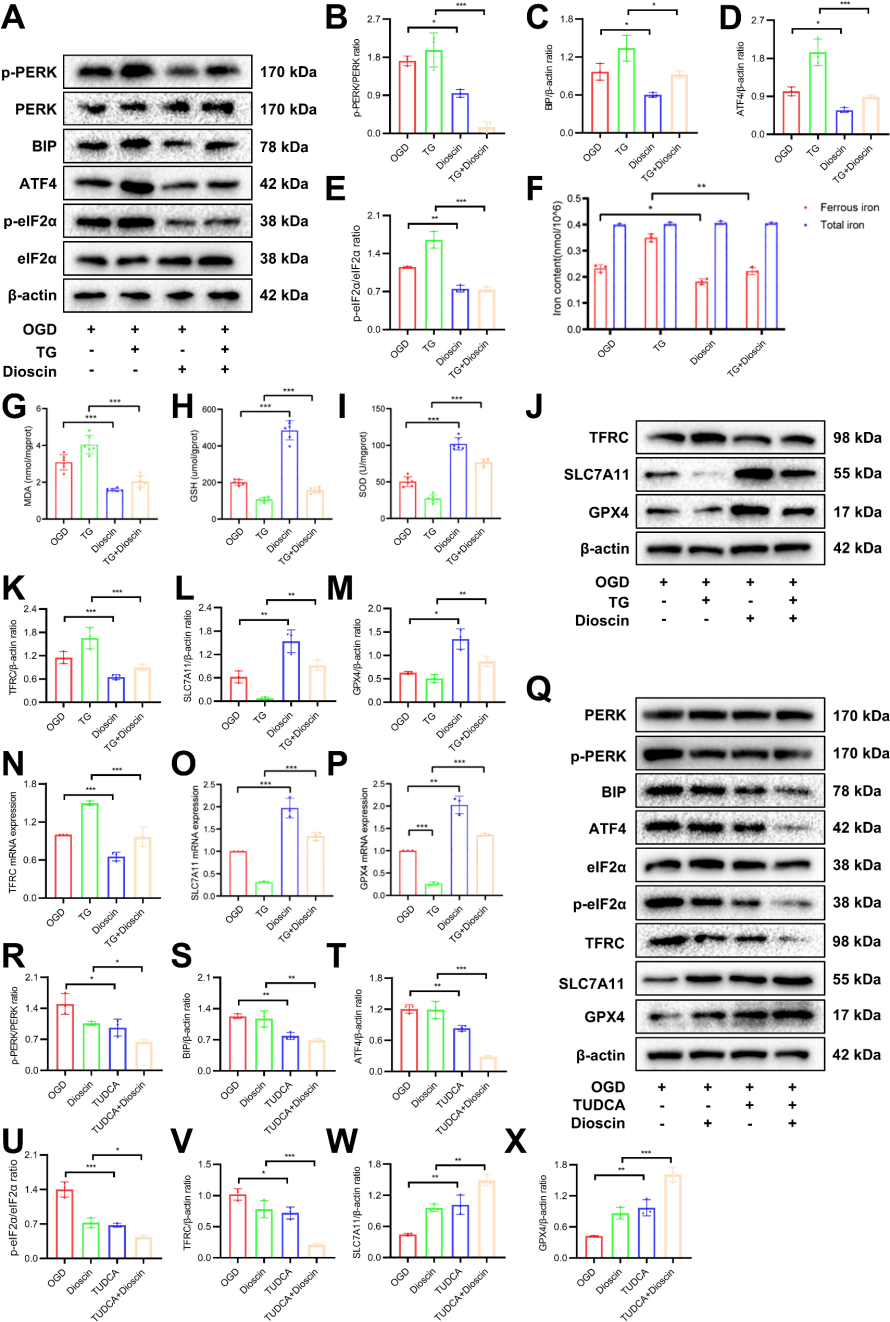
Dioscin pretreatment inhibited ferroptosis via suppressing ER stress in vitro. (**A**) Representative Western blot images of ER stress markers in H9C2 cells subjected to OGD, with and without TG (ER stress inducer). (**B**-**E**) Quantification of PERK phosphorylation (**B**), GRP78/BIP (**C**), ATF4 (**D**), and eIF2α phosphorylation (**E**) expression (*n* = 3). (**F**) Fe^2+^ and total iron content (*n* = 6). (**G**-**I**) MDA (**G**), SOD (**H**), and GSH (**I**) levels (*n* = 6). (**J**) Representative Western blot images of ferroptosis markers in H9C2 cells subjected to OGD, with and without TG (ER stress inducer) (*n* = 3). (**K**-**M**) Quantification of TFRC (**K**), SLC7A11 (**L**), and GPX4 (**M**) proteins (*n* = 3). (**N**-**P**) mRNA levels of TFRC (**N**), SLC7A11 (**O**), and GPX4 (**P**) were detected by qPCR (*n* = 3). (**Q**) Representative Western blot images of ER stress and ferroptosis markers in H9C2 cells subjected to OGD, with and without TUDCA (ER stress inhibitor). (**R**-**X**) Quantification of PERK phosphorylation (**R**), GRP78/BIP (**S**), ATF4 (**T**), eIF2α phosphorylation (**U**), TFRC (**V**), SLC7A11 (**W**), and GPX4 (**X**) proteins (*n* = 3). ^*^P < 0.05, ^**^P < 0.01, ^***^P < 0.001

To further investigate whether ER stress is associated with the protective effect of Dioscin pretreatment against ferroptosis in cardiomyocytes after OGD, we utilized the ER stress inducer Thapsigargin (TG). There was a higher stress index of endoplasmic reticulum in the OGD group with TG compared to the OGD group without TG. According to the Western blot results, those in the OGD with TG treatment significantly increased the levels of the phosphorylation levels of PERK and eIF2α proteins as well as ATF4 and GRP78/BIP protein expression (Fig. 6A-E). As mentioned previously, Dioscin pretreatment protected cardiomyocytes against ER stress induced by OGD. The content of ferrous iron also increased after TG was added, and the content of ROS increased after Fe^2+^ participated in the Fenton reaction, which aggravated ferroptosis (Fig. 6F). SOD and GSH were consumed more after TG was added, while MDA products representing lipid peroxidation were produced more (Fig. 6G-I). Subsequently, we compared the levels of ferroptosis biomarkers. The results showed that TG inhibited the anti-ferroptosis effect of Dioscin pretreatment (Fig. 6J-P). Therefore, Dioscin pretreatment could reduce OGD-induced cardiomyocyte ferroptosis by inhibiting ER stress. In addition, we further confirmed this result using Tauroursodeoxycholic acid (TUDCA), an ER stress inhibitor (Fig. 6Q-X).

## Discussion

In this study, we demonstrated that Dioscin pretreatment inhibits the ER stress response and cardiomyocyte ferroptosis in mice with MI. In the presence of MI, Dioscin pretreatment can relieve cardiac dysfunction and myocardial damage. Dioscin pretreatment inhibited ER stress through PERK- eIF2α-ATF4 signaling, reducing ferroptosis.

MI is a serious cardiovascular disease, primarily caused by myocardial ischemia and hypoxia, which can lead to complications like heart failure or arrhythmia (Collaboration 1994; Lloyd-Jones et al. 2009; Yu et al. 2023). As a result of the initial stage of hypoxia, myocardial cells die, causing remodeling of the ventricular structure and impairing the heart’s pumping function during the recovery period, which eventually leads to heart failure (Boden et al. 2007). Since the 1960s, Dioscin has been used as the main active ingredient in traditional herbal products for the treatment of coronary heart disease in the former Soviet Union and China. A growing body of research indicates that Dioscin has a wide range of pharmacological activities in heart disease (Kong et al. 2021; Zhang et al. 2022c). In this study, using Dioscin pretreatment in MI mice attenuated the myocardium injury caused by MI. Dioscin pretreatment significantly improved the survival rate of mice post-MI surgery. Histological assessments and cardiac functional parameters, including myocardial infarct size, leukocyte infiltration, and cardiac contractile function, highlighted the protective effects of Dioscin pretreatment. This was further supported by the reduction in cardiac injury biomarkers (cTnT, CK-MB, and LDH) upon Dioscin pretreatment.

On the other hand, a microenvironment of oxidative stress is created in the ischemic area by ROS produced (Cao et al. 2022; Li et al. 2022). Myocardial ischemia is thus primarily concerned with oxidative damage, which is also closely related to ferroptosis (Mei et al. 2022). Ferroptosis, on the other hand, is a non-apoptotic method of cell death (Guo et al. 2022; He et al. 2022). Ferroptosis occurs when excessive lipid peroxidation and iron overload occur within the cell (Komai et al. 2022). A majority of its morphological features involve mitochondrial changes, including mitochondrial shrinkage, increased mitochondrial membrane density, crista destruction, and rupture of the outer membrane (Shaghaghi et al. 2022). The effectiveness of ferroptosis inhibitors in the human body is greatly limited by their toxicity, instability, and short half-life (Li et al. 2021; Shaghaghi et al. 2022). It is urgent to develop non-toxic, long-acting inhibitors of ferroptosis (Chen et al. 2021c; Chen 2022). Ferroptosis has been linked to myocardial injury in increased studies, but its exact effects on the ischemic heart remain unclear (Luo et al. 2021). Ferroptosis aggravates myocardial oxidative injury, and targeting ferroptosis to prevent cardiac cell death was an effective cardioprotective strategy (Chen et al. 2021b; Hu et al. 2021). Previous studies have shown that Dioscin reduces ferroptosis in cisplatin-induced acute kidney injury (Wang et al. 2022). In this work, the pretreatment of Dioscin reversed the phenotypes of GSH inhibition, MDA production, lipid ROS production, and iron deposition in MI mice. TFRC expression was reduced and GPX4 and SLC7A11 were increased in MI mice, but Dioscin pretreatment rescued the expression. Transferrin (serum transferrin or lactoferrin) mediates iron uptake via TFRC (Kim et al. 2023). If TFRC is overexpressed, then iron uptake is increased and cells subsequently become more susceptible to ferroptosis (Yi et al. 2022). GPX4 is a selenocysteine-containing enzyme that inhibits lipid peroxidation in membranes and is known as a key inhibitor of ferroptosis (Chen et al. 2021a). SLC7A11 is an amino acid transporter protein that is specific and is a key regulator of ferroptosis (Koppula et al. 2021). The down-regulation of SLC7A11 can indirectly inhibit the activity of GPX4 by inhibiting the cysteine metabolic pathway, leading to the decrease of intracellular cystine levels and the depletion of GSH biosynthesis, which in turn leads to the accumulation of lipid peroxides and eventually induces ferroptosis in cells (Zhang et al. 2022a). The regulation of these proteins confirmed the ability of Dioscin pretreatment to alleviate the ferroptosis that occurs in MI mice. In vitro experiments with H9C2 cells subjected to OGD confirmed pretreated-Dioscin’s protective effects against OGD-induced injury. Dioscin pretreatment reduced ferrous iron accumulation, restored cellular antioxidant levels, and modulated ferroptosis-related gene and protein expression, paralleling its effects observed in the MI model.

Extreme hypoxia will induce ER stress, resulting in unfolded protein response. Unfolded proteins will competitively bind with the chaperone GRP78/BIP originally attached to PERK, IRE1, and ATF6. The original three sites will be activated due to the loss of GRP78, and ER stress will be initiated. IRE1 and ATF6 downstream may regulate ER stress through negative feedback into the nucleus. This study delved into the impact of Dioscin pretreatment on ER stress during MI. Elevated ER stress markers observed in the MI model were partly mitigated by Dioscin pretreatment. The downregulation of the phosphorylation levels of PERK and eIF2α proteins as well as ATF4 and GRP78/BIP protein expression in Dioscin-pretreated mice indicates its potential in suppressing ER stress. There is close cross-talk between ferroptosis and ER stress signaling in recent research (Kim et al. 2022; Liang et al. 2022; Zhang et al. 2022b). In particular, ER stress disrupts Ca^2+^ homeostasis, leading to mitochondrial calcium overload and ROS production (McGrath et al. 2021; Sha et al. 2021). ER stress may be further increased by ferroptosis inducers, which may promote the cystine-glutamate antiporter system, Xc-, through the unfolded protein response (Wang et al. 2020). Ferroptosis can also be triggered by ROS, which are also primary promoters of ER stress (Nechushtai et al. 2020). Our study further elucidated the link between ER stress and pretreated-Dioscin’s protective role against ferroptosis in OGD-induced cardiomyocyte injury. Dioscin pretreatment demonstrated a capacity to suppress ER stress induced by OGD, thus inhibiting subsequent ferroptotic processes. This was substantiated by experiments utilizing an ER stress inducer (TG), which compromised pretreated Dioscin’s anti-ferroptosis effects, while the ER stress inhibitor (TUDCA) enhanced these effects, emphasizing the interplay between ER stress and ferroptosis in this context. Our study showed Dioscin pretreatment inhibited ER stress through PERK-eIF2α-ATF4 signaling, reducing ferroptosis.

It is noteworthy that many other cells, such as fibroblasts and macrophages, are also involved in the pathophysiologic process of MI. However, our studies have mainly focused on cardiomyocytes and have not extensively investigated the effects of Dioscin pretreatment on other cells. Therefore, there is a need to further investigate the effects of Dioscin pretreatment on these cells under the pathologic conditions of MI. Recent findings have highlighted the role of TRIM29 in promoting cardiac injury through the activation of the PERK-mediated ER stress pathway in viral myocarditis (Wang et al. 2024). Given that our study demonstrated that Dioscin alleviates PERK-mediated ER stress in both in vivo and in vitro models of MI and inhibits ferroptosis in cardiomyocytes, this may involve the downregulation of TRIM29. Future studies should investigate whether Dioscin pretreatment affects TRIM29 expression and its role in regulating ER stress in MI models, providing further insights into the molecular mechanisms by which Dioscin protects the heart from injury. Despite these limitations, the present study demonstrated that Dioscin pretreatment attenuates myocardial injury in MI by inhibiting ferroptosis via PERK-eIF2α-ATF4. The finding provides new evidence that Dioscin pretreatment may have preventive effects on MI by targeting ferroptosis.

## Data Availability

All data included in this study are available upon request by contact with the corresponding author.
